# Gender Difference in Food Choice and Eating Practice and Their Association with Health among Students of Kathmandu, Nepal

**DOI:** 10.1155/2022/2340809

**Published:** 2022-08-25

**Authors:** Maginsh Dahal, Alisha Basnet, Sudip Khanal, Kushalata Baral, Smriti Dhakal

**Affiliations:** ^1^School of Public Health, Nanjing Medical University, Nanjing, China; ^2^Department of Public Health, Nobel College, Pokhara University, Kathmandu, Sinamangal, Nepal; ^3^Department of Biostatistics and Epidemiology, University of North Texas Health Science Centre, Fort Worth, USA

## Abstract

**Background:**

Our eating practice is generally based on the food we choose to eat. The selection of unhealthy food, high cost of healthy food items, and easy availability of fast food may have negative impact on our health and eating behaviour. This study aims to access the gender difference in food choice and eating practice and their association with health among students in Kathmandu, Nepal.

**Methods:**

A cross-sectional descriptive study was conducted among 385 randomly selected undergraduate BBA (Bachelor in Business Administration) students of Tribhuvan University in Kathmandu by using semistructured self-administered questionnaire. The questionnaire included sociodemographic characters, health status, behaviour factors, eating practice, and food choice which were measured using Food Choice Questionnaire (FCQ). The data was analysed in SPSS. Frequency, percentage, mean, and standard deviation were calculated, and chi-square test and logistic regression were used to measure the association between two variables.

**Results:**

The study is comprised of 50.4% female and 49.6% male with mean ages of 20.04 and 20.75, respectively. A gender difference was observed in food choice but no gender difference was observed in eating practice. There was no significant association of food choice and eating practice with health. However, food choice and eating practice showed an association with the current living status of the respondents. Sensory appeal and health were the most important food choice motives among males and females, respectively.

**Conclusion:**

The study concluded that no gender difference was observed in food choice. However, gender difference was observed in eating practice. There was no association of food choice and eating practice with health.

## 1. Introduction

Food choice refers to the process of deciding what to eat, which varies from person to person and is influenced by a variety of factors such as health, price, and mood. The choice we make on food determines which nutrient enters our body [1]. The food choices are made every day, every time, from what vegetables we prefer with our meal to what food we choose to eat in canteen or in restaurant [2]. We make choices according to our mood, preference, taste, quality, quantity, income, situation, cravings, or eating behaviours [2]. Food choice is important because it may represent the motive for the usual purchase of a food or preparation of a meal [2].

Eating practice is the way of how persons eat and what types of food they eat and when. Our eating practice is usually based on the food we choose to eat. It differs from person to person. The type of food we choose and buy, the way we prepare food, and the way we eat food define how well we live [3]. Eating practices are influenced by a variety of factors, including lifestyle factors and mental health state [3]. Our eating practice determines our health status. Many health conditions are caused by the food we eat such as obesity, high cholesterol, gastritis, and heart disease [4]. Eating practices have been identified as one of the factors influencing the global overweight and obesity epidemic [3].

With the transition from secondary school to bachelor's level, the independency increases and students are constantly challenged to make healthy food selections [5]. Students face a new environment for meal preparation, planning, choosing food, and eating as they move to their college life [6]. Their eating habits usually shape or change during this period [7]. So, they may face difficulty in regular eating behaviour and this may not always lead to healthy food choice which can cause significant health problems. Poor eating habits among young adults are major public health concern. The selection of unhealthy food, high cost of healthy food items, and easy availability of fast food may have negative impact on health and eating behaviour of undergraduate students [8]. Similarly, male students are more likely to eat fast food, whereas they are less likely to have breakfast and prepare their own food compared to female students [9, 10]. Female students consume adequate quantities of fruits, vegetables, and milk substances compared to male students [11].

Although eating habits are major determinant of health status, very few studies have been conducted on the food choice and eating practice in Kathmandu Metropolitan City. Furthermore, no similar studies have been performed among students in Nepal based on their gender, religion, and their current living status. As most of the students live away from their family in Kathmandu, it is important to know if these factors affect their food choice and eating practice. Also, religion factor makes restrictions to choose and eat certain food items. Thus this research tries to identify the gender difference in food choice and eating practice and their association with health among students in Kathmandu, Nepal.

## 2. Methods

The descriptive cross-sectional study using quantitative methods was conducted among the undergraduate BBA students of four different colleges affiliated to TU (Tribhuvan University) of Kathmandu. The sample size was 385 and it was calculated using 50% prevalence with confidence limit of 95% and 5% error using the following formula:

(1) $$\begin{array}{c} n=\frac{\left[Z^{2}x\left(p\right)x\left(1-p\right)\right]}{e^{2}}. \end{array}$$

Among the total 16 BBA (Bachelor in Business Administration) colleges affiliated to TU within Kathmandu, 4 colleges were selected using lottery method. The total number of students who were reading BBA from the selected colleges were 1186 which was the sampling frame. Then, Probability Proportional to Size (PPS) was used to calculate the required number of respondents from each college and students were selected using random table. Students who were aged 18 years and above and were willing to participate were included in the study. Those students who refused to participate were replaced by randomly selected new participants.

Semistructured self-administered questionnaire was used to collect the data. The questionnaire included sociodemographic characters, health status, behaviour factors, eating practice, and food choice of the students. The health status of the respondents was measured using certain questionnaires including their Body Mass Index, sick frequency, health problems, medications, and food related health problem within 3 months such as diarrhoea, food poisoning, cholera, typhoid, and bloating. The Body Mass Index was calculated to measure the respondents' weight with respect to their height by using the formula BMI = kg/m^2^, where kg is a person's weight in kilogram and m^2^ is the square of their height in meters. The behavioural factors such as smoking, alcohol consumptions, additional food intake, food items avoided, and fasting were included as covariables. The students' food choice was measured using Food Choice Questionnaire (FCQ) which was developed by Steptoe. et al [12]. FCQ measures the impact of both health-related and non-health-related factors on people's food choices. It contains 36 items designed to assess the importance of nine factors, health, mood, convenience, sensory appeal, natural content, price, weight control, familiarity, and ethical concern, among which 20 items were included. The scores for each item ranged as 1 (not important at all), 2 (a little important), 3 (moderately important), and 4 (very important). The scores that contributed to each factor were calculated by the average score of the items, so the scale scores were between 1 and 4. Similarly, students' eating practice was based on a scale of two (“1”, and “0”). A score of “1” was awarded for good eating practice. A score of “1” was awarded for eating practice where the person eats three or more than three times in a day, who regularly eats breakfast, vegetables, fruits, and dairy products, who regularly drinks 1 or more liter of water every day, and who rarely or never eats sweets, bakery items, and junk foods or eats outside home, while a score of “0” was awarded for poor eating practice, where the person eats only two times in a day, who does not regularly eat breakfast, vegetables, fruits, and dairy products, who drinks less than a liter of water in a day, and who often eats sweets, bakery items, and junk foods or eats outside home, and a total score was computed for each respondent.

The question was developed in simple and clear English language. Validity of the instrument was obtained from literature review. Random error was reduced by selecting adequate sample size. Pretesting of questionnaires was done among 38 individuals to ensure reliability. Ethical approval was obtained from the Institutional Review Committee of Nobel College. The college authorities were contacted for permission to conduct the study. Participants were briefed about the study objectives and techniques of filling the questionnaire, and written consent was taken from each participant before collecting the data. Participation in this study was fully voluntary and privacy and confidentiality of the collected information were ensured. Each and every filled questionnaire was rechecked on the spot.

The data entry was done in Epi-Data and the data were analysed using the IBM SPSS version 26. The data analysis was carried out using descriptive statistics and inferential statistics. Under descriptive analysis of data, frequency and percentage were calculated to find out the sociodemographic characteristics, health status, behavioural factors, and eating practices of the respondents. Additionally, mean and standard deviation were also calculated to measure the average score and the variation in food choice motives, and the chi-square test was used to measure the association between dependent and independent variables. Variables that were found to be statistically significant (*p* < 0.05) during chi-square test were then further analysed using logistic regression model in multivariate analysis. Hosmer and Lemeshow test was used to test the goodness of fit for the regression model. Adjusted odds ratio with 95 percent CI and *p* value was calculated.

## 3. Results

Table 1 presents the descriptive statistics for sociodemographic characters, health status, and behavioural factors of the respondents. It shows that 50.4% of the respondents were female with the mean age of 20.04 years and standard deviation of 1.195 years. The majority of the respondents, that is, 89.5% of males and 92.8% of females, were Hindu. The majority of them were Chhetri (30.9% of males and 37.1% of females). Most of the respondents, that is, 58.6% of males and 82% of females, were living with family.

Moving towards the health status of the respondents in terms of male and female, the majority of the respondents (85.3% of males and 74.7% of females) had a normal body weight. About 37.2% of males and 57.2% of females get sick sometimes. Nearly 59.7% of males and 41.2% of females did not have any health problem due to the food they ate in the last 3 months. Apart from the health problem raised from the food ate in the last 3 months, 57.6% of males and 71.1% of females had other health problems such as gastritis, underweight, tooth decay, overweight, hypertension, heart diseases, hypotension, and PCOS. Majority of the respondents (94.8% of males and 97.4% of females) were not under any type of medications.

The behavioural factors of male and female respondents demonstrate that none of the females smoke, while about 33% of males smoke. About 40.3% of males and 78.9% of females did not drink alcohol. More than half of the respondents, that is, 57.6% of males and 56.7% of females, were not avoiding any food items. However, more than half of the respondents, that is, 53.9% of males and 52.6% of females, were taking additional food items including tea or coffee, junk foods, sweets/chocolates, salt, and sugar. Most of the respondents, that is, 72.3% of males and 53.1% of females, were not involved in fasting.

In Table 2, various responses were reflected by respondents to the question related to the food eating practice. It shows that 36.2% of males and 46.4% of females eat three times per day. Around 59.2% of male and 62.4% of female respondents did not eat breakfast regularly for various reasons including being unable to eat early in the morning, not having enough time for breakfast, and sometimes not having food at home. Majority of the respondents (59.2% of males and 76.8% of females) eat vegetables every day. About two-thirds of respondents (66% of males and 62.4% of females) consume fruits sometimes. Majority of the respondents, that is, 58.1% of males and 63.4% of females, consume meat 1–3 days per week. Most of the respondents (58.1% of males and 42.3% of females) eat dairy products 1–3 days per week. About 61.3% of males and 62.4% of females consume sweets and bakery items sometimes. In terms of junk food (foods that are easily available, inexpensive, high in calories, and low in nutrients) intake, it was discovered that more than half of the respondents (55% of males and 54.6% of females) consume junk foods sometimes. It was also found that majority of the respondents (62.9% of males and 71.1% of females) eat at cafes and restaurants or outside home sometimes. Around 34.1% of males and 27.3% of females drink 2 liters of water per day. In the domain, more than half of the respondents, 54.5% of males and 58.8% of females, were satisfied with their eating pattern. Majority of the respondents, that is, 84.3% of males and 87.1% of females, stated that they do not skip meals or any food items. However, 15.7% of males and 12.9% of females skipped meals or food items for a variety of reasons, including intermediate fasting, weight control, lack of time, and laziness to cook.

Table 3 represents the food choice motives of the respondents with reference to gender. The questions used to measure food choices were divided into 9 food choice motives: health, mood, price, convenience, sensory appeal, natural content, weight control, familiarity, and ethical concern. The result shows that sensory appeal ranked first among males' food choice motives, while health ranked first among females' food choice motives. Similarly, among the males' food choice motive, health ranked second, convenience third, mood fourth, familiarity fifth, price sixth, natural content seventh, ethical concern eighth, and weight control ninth, whereas, among the female's food choice motive, sensory appeal ranked second, convenience third, familiarity fourth, mood fifth, ethical concern sixth, weight control seventh, natural content eighth, and price ninth.

Table 4 shows the association of food choice and eating practice with sociodemographic characters, health status, and behavioural factors of the respondents. The gender was strongly associated with food choice having *p* value ≤ 0.001^*∗*^. The religion was significantly associated with food choice having *p* value = 0.031^*∗*^. The current living status of the respondents was significantly associated with food choice and eating practice having *p* value = 0.018^*∗*^ and ≤0.001^*∗*^, respectively.

The smoking factor was significantly associated with food choice and eating practice having *p* value = ≤0.001^*∗*^ and 0.018^*∗*^, respectively. However, the alcohol was significantly associated with food choice having *p* value = 0.013^*∗*^ but was not significantly associated with eating practice having *p* value = 0.236. It is shown that avoiding food items was not significantly associated with food choice having *p* value = 0.930 but was significantly associated with eating practice having *p* value = 0.034^*∗*^.

Table 5 shows the logistic regression for good food choice and good eating practice. It states that females were 1.738 times more likely to have good food choice compared to males. Non-Hindu were 0.463 times less likely to have good food choice compared to Hindu. Similarly, respondents who were living with family were 1.387 time more likely to have good food choice compared to respondents who were not living with family. Respondents who smoke were 0.612 times less likely to have good food choice compared to those who did not smoke. Furthermore, respondents who drank alcohol were 0.888 times less likely to have good food choice compared to those who did not drink alcohol. Similarly, the respondents who were living with family were 2.062 times more likely to have good eating practice compared to respondents who were not living with family. Respondents who smoke were 0.546 times less likely to have good eating practice compared to those who did not smoke. Similarly, respondents who avoided food items were 1.670 time more likely to have good eating practice compared to those who did not avoid food items.

## 4. Discussion

This study aims to access the gender difference in food choice and eating practice and their association with health among undergraduate students of Kathmandu Metropolitan City.

### 4.1. Difference in Food Choice

The study reveals the significant association between the gender and food choice having *p* value = 0.000^*∗*^ but no significant association was observed between gender and eating practice (*p* value = 0.575). In the previous study conducted among the guardians of Trilok Academy Kathmandu with reference to gender, no significant association between the gender and food choice was observed [13]. The differences in the study population might contribute to the different results in both studies. However, in another study done in a large sample of university students from 23 different countries, 50% gender difference was observed in food choice and 40% gender difference was observed in eating practice [14]. The study said this might be partly due to women's greater weight control involvement and partly due to their strong beliefs in healthy eating [14].

Apart from that, this study also states that health was the most important food choice motive among females with the mean average rating of 3.34 ± 0.64, while sensory appeal was the most important food choice motive among males with the mean average rating of 3.12 ± 0.67 on a 4-point rating scale. The study also adds that weight control was the least important food choice motive among males with the mean average rating of 2.41 ± 0.66, while females rated price as the least important food choice motive with the mean average rating of 2.64 ± 0.8. In a similar study conducted in Malaysia among husbands and wives, health was the most important food choice motive among wives with the mean average rating of 4.49 ± 0.58, while religion was the most important food choice motive among husbands with the mean average rating of 4.56 ± 0.59 on a 5-point rating scale. Similarly, both husbands and wives rated familiarity as the least important food choice motive with the mean average ratings of 3.07 ± 0.77 and 3.08 ± 0.66, respectively [8]. Furthermore, in another study conducted in Kathmandu, both the males and females rated health as the most important food choice motive with the mean average ratings of 3.04 and 2.96, respectively, while weight control was the least important food choice motive among both males and females with the mean average ratings of 2.35 and 2.31, respectively [13]. It seems that people were more health conscious about what they eat and less conscious about the weight control. This might be because almost three-fourths of the respondents have normal body weight in this study.

### 4.2. Difference in Eating Practice

The eating practice seems average in this study. However, the percentage of the students who skip their breakfast was quite high, that is, 59.2% of males and 62.4% of females. These findings are consistent with similar study conducted among university students in Selangor, Malaysia, which reported that 34.4% of males and 47.3% of females regularly take breakfast, while 59% of males and 52.8% of females skip their breakfast [15]. Majority of the respondents in this study reported that they cannot eat early in the morning and have no time for breakfast. The previous study conducted in Kuala Lumpur suggested that the reasons for skipping the breakfast were oversleeping, lack of time, lack of appetite, and inability to eat early in the morning [16].

This study reveals that 76.8% of females and 59.2% of males consume vegetables regularly, whereas 28.9% of females and 19.9% of males consume fruits regularly. The fruits and vegetables intake among females seems to be better than that among males. A similar finding was reported by another study conducted among Oregan State University students which showed that female students were engaged in various healthful eating habits including higher consumption of fruits and vegetables as compared to male students [17]. A study conducted among urban college freshmen in USA states that the intake of fruits and vegetables decreased with the beginning of the college and with the increase in consumption of the fast foods [18]. Apart from that, this study also reveals that majority of the males, that is, 27.2%, regularly eat outside home compared to the females (16%). This might be because of the easy availability of the fast food restaurants and cafes or because majority of the male students were not living with the family compared to female students.

The study says that 58.6% of males and 82% of females were living with family, while the remaining 41.4% of males and 18% of females were living with friends or in hostel or living independently. Similarly, the study also showed the significant association between current living status of the students with food choice and eating practice of the respondents. Students who were living with family were 1.387 times more likely to have good food choice and 2.062 times more likely to have good eating practice compared to those who were not living with family. The previous study which was conducted in Greece states that the students who were living at home had no change in their eating practice but students living away from home had some changes including decrease in the weekly consumption of fruits and vegetables and increase in sugar, wine, and alcoholic beverage intake [19]. This might be because the students living away from home organize the shopping and preparation of food on their own, which can have a negative impact on their eating habits [19].

Similarly, in this study, the meat intake was found to be more among males compared to females, whereas the junk food, sweets, and bakery items intake was found to be more among females compared to males. The study reveals that around 11.5% of males and 4.6% of females regularly consume meat. Around 30.4% of females and around 16.2% of males regularly consume junk food. Also, 11.8% of females and 7.8% of males regularly eat sweets and bakery items. This finding is similar to that of a previous research performed among college students in Kuwait which tells that 48.4% of males and 28.8% of females regularly consume meat and 51.3% of females and 40.4% of males regularly consume potato chips and fatty salty snacks, whereas 52.5% of females and 39.9% of males regularly eat sweets such as cakes and chocolates [20].

### 4.3. Health Status of the Respondents

In this study, majority of the respondents had normal body weight. The normal body weight was more prevalent among males (85.3%) as compared to females (74.7%). Similarly, overweight was more prevalent among males (7.9%) as compared to females (5.7%). The majority of females, 19.6%, were underweight compared to 6.8% of males. These findings are consistent with another study conducted among Lebanese students which reported that majority of females, 76.8%, had normal body weight as compared to males (49%), whereas overweight was more common among males by 37.5% compared to females, that is, 13.5%. Underweight was more prevalent among females (6.4%) as compared to males (1%) [21]. Another study conducted among college students in Kuwait said that 44.8% of males and 62.2% of females had normal body weight, while 28.7% of males and 19.9% of females were overweight. Furthermore, 5.8% of males were underweight as compared to females (2.8%) [20]. The previous study suggested that the high prevalence of underweight among females might be due to the desire to have a leaner and thinner body and more negative attitude towards their bodies [22].

The study shows that more than half of the females, that is, 58.8%, and 40.3% of males had health problem from the food they ate such as bloating, food poisoning, diarrhoea, typhoid, cholera, and others including anaemia, which is similar to a previous study about food-borne disease outbreaks conducted in USA, where food borne-associated diseases were slightly more likely to be in females (54%) than in males (46%) [23]. This might be because females were more attracted to the junk food, sweets, and bakery items as compared to males in this study.

### 4.4. Behaviour of the Respondents

The smoking and alcohol factors showed a significant association with the food choice having *p* value = 0.001 and 0.013, respectively. Also, the smoking factor showed significant association with the eating practice having *p* value = 0.018. The study tells that the respondents who smoke were 0.546 times less likely to have good eating practice compared to those who did not smoke. In this study, 33% of males were smokers, while none of the females were smokers. Similarly, 59.7% of males and 21.1% of females had habit of drinking alcohol. This finding is similar to that of another study conducted among Kuwait University students which states that smoking was prevalent among 32.4% of males, while only 1% of females were smokers [20]. Another study conducted among Lebanese student states that the alcohol intake and smoking were not a common practice among the students [21]. The percentages of males who smoke and drink alcohol were high, and that might be because of the peer pressure as majority of the males were not living with family but were living with friends or in hostel or independently.

The study reveals that majority of the respondents, that is, 72.3% of males and 53.1% of females were not fasting, while 27.7% of males and 46.9% of females were fasting. A similar study conducted among the university students at Southern Nigeria states that 71.2% of males and 69.6% of females reported not being on diet, which showed that dieting was not the common practice among the students [3].

## 5. Conclusion

This study concluded that there was a gender difference in food choice but no gender difference was observed in eating practice. However, the food choice and eating practice were dependent on the current living status of the students. There was no significant association of food choice and eating practice with health. Sensory appeal and health were the most important food choice motives among males and females, respectively. Majority of the respondents had normal body weight but still overweight was more prevalent among males, while underweight was prevalent among females. This study helps to bring the positive change in food and health behaviour among university students and also adds more knowledge to the health educators in developing strategies and implementing interventions. The findings may also be used as a baseline for measuring the effectiveness of possible interventions.

### 5.1. Limitations

The study was limited for fixed duration of time and was limited among undergraduate BBA students under TU within Kathmandu Metropolitan City. So, the scenarios of the whole population may not be generalized. Furthermore, self-administered technique was adapted for data collections which were highly dependent on the respondents' memory, honesty, and truthfulness in answering the questions. Also, the weight and height of the respondents were not measured but were dependent on the respondents' memory and assumption. Therefore, the results may not reveal the actual Body Mass Index of the respondents. Notwithstanding these limitations, the results contribute to, and take forward, the issue of gender differences in food choice and eating practice. More similar studies are needed in the general population as well as in other areas such as age, ethnicity, income, and education level.

## Figures and Tables

**Table 1:** Sociodemographic characteristics, health status, and behavioural factors of the respondents (*n* = 385).

Variable	Male (*n* = 191) (49.6%)	Female (*n* = 194) (50.4%)
*Age*		
20 and under	89 (46.6)	140 (72.2)
21 and above	102 (53.4)	54 (27.8)
Mean ± SD	20.75 ± 1.414	20.04 ± 1.195

*Religion*		
Hindu	171 (89.5)	180 (92.8)
Buddhist	7 (3.7)	6 (3.1)
Christian	8 (4.2)	7 (3.6)
Muslim	5 (2.6)	1 (0.5)

*Ethnicity*		
Brahmin	60 (31.4)	68 (35.1)
Chhetri	59 (30.9)	72 (37.1)
Janajati	64 (33.5)	47 (24.2)
Others	8 (4.2)	7 (3.6)

*Currently living status*		
Living independently	19 (9.9)	7 (3.6)
Living with family	112 (58.6)	159 (82)
Living with friends	32 (16.8)	8 (4.1)
Student residence (hostel)	28 (14.7)	20 (10.3)

*Family's monthly income (Nepalese rupees)*		
Below 15,000	5 (2.6)	0
15,000–29,000	21 (11)	22 (11.3)
30,000–49,000	65 (34)	58 (29.9)
50,000–99,000	72 (37.7)	70 (36.1)
1,00,000 and more	28 (14.7)	44 (22.7)

*BMI*		
Underweight	13 (6.8)	38 (19.6)
Normal	163 (85.3)	145 (74.7)
Overweight	15 (7.9)	11 (5.7)

*Sickness frequency*		
Very frequently	11 (5.8)	18 (9.3)
Sometimes	71 (37.2)	111 (57.2)
Rarely	103 (53.9)	64 (33)
Never	6 (3.1)	1 (0.5)

*Food related health problem within 3 months*		
Yes	77 (40.3)	114 (58.8)

*Other health problems*		
Yes	110 (57.6)	138 (71.1)

*Medications*		
No	181 (94.8)	189 (97.4)

*Smoke*		
No	128 (67)	194 (100)

*Alcohol*		
Yes	114 (59.7)	41 (21.1)

*Food items avoided*		
No	110 (57.6)	110 (56.7)

*Additional food intake*		
Yes	103 (53.9)	102 (52.6)

*Fasting*		
No	138 (72.3)	103 (53.1)

**Table 2:** Eating practice of respondents (*n* = 385).

Variable	Male (*n* = 191) (%)	Female (*n* = 194) (%)
Eat per day		
Two times	14 (7.3)	4 (2.1)
Three times	69 (36.2)	90 (46.4)
Four times	64 (33.5)	69 (35.5)
More	44 (23)	31 (16)

Eat breakfast regularly		
No	113 (59.2)	121 (62.4)

Eat vegetables per week		
Every day	113 (59.2)	149 (76.8)
1–3 days per week	37 (19.4)	19 (9.8)
4–6 days per week	41 (21.5)	26 (13.4)

Eat fruits in diet		
Regularly	38 (19.9)	56 (28.9)
Sometimes	126 (66)	121 (62.4)
Rarely	23 (12)	16 (8.2)
Never	4 (2.1)	1 (0.5)

Eat meat per week		
Every day	22 (11.5)	9 (4.6)
1–3 days per week	111 (58.1)	123 (63.4)
4–6 days per week	44 (23.1)	24 (12.4)
Never	14 (7.3)	38 (19.6)

Eat dairy products per week		
Every day	48 (25.1)	78 (40.2)
1–3 days per week	111 (58.1)	82 (42.3)
4–6 days per week	17 (8.9)	19 (9.8)
Never	15 (7.9)	15 (7.7)

Eat sweets and bakery items in diet		
Regularly	15 (7.8)	23 (11.8)
Sometimes	117 (61.3)	121 (62.4)
Rarely	52 (27.2)	43 (22.2)
Never	7 (3.7)	7 (3.6)

Consume junk foods (Lays, Kurkure, biscuits, chips, noodles, chatpate, panipuri, etc)		
Regularly	31 (16.2)	59 (30.4)

Sometimes	105 (55)	106 (54.6)
Rarely	41 (21.5)	22 (11.4)
Never	14 (7.3)	7 (3.6)

Eat at cafes and restaurants or outside home		
Regularly	52 (27.2)	31 (16)
Sometimes	120 (62.9)	138 (71.1)
Rarely	19 (9.9)	25 (12.9)

Drink water per day		
1–5 glasses	35 (18.3)	64 (33)
1 liter	43 (22.5)	49 (25.3)
2 liters	65 (34.1)	53 (27.3)
More	48 (25.1)	28 (14.4)

Satisfied with eating pattern		
Yes	104 (54.5)	114 (58.8)

Skipped meal or food items		
No	161 (84.3)	169 (87.1)

**Table 3:** Food choice motives with reference to gender.

Food choice motives	Male			Female		
	Mean	Standard deviation	Rank	Mean	Standard deviation	Rank
Health	3.05	0.76	2	3.34	0.64	1
Mood	2.68	0.77	4	2.85	0.77	5
Price	2.57	0.76	6	2.64	0.8	9
Convenience	2.7	0.74	3	2.97	0.8	3
Sensory appeal	3.12	0.67	1	3.26	0.68	2
Natural content	2.54	1.05	7	2.71	1.1	8
Weight control	2.41	0.66	9	2.76	0.68	7
Familiarity	2.58	0.7	5	2.91	0.81	4
Ethical concern	2.54	0.69	8	2.76	0.7	6

**Table 4:** Association between food choice and eating practice with sociodemographic characteristics, health status, and behavioural factors of the respondents.

Variables	Food choice		Chi-square value (*χ*^2^)	*p* value	Eating practice		Chi-square value (*χ*^2^)	*p* value
	Poor food choice *n* (%)	Good food choice *n* (%)			Poor practice *n* (%)	Good practice *n* (%)		
*Age (in years)*								
20 and below	112 (48.9)	117 (51.1)	3.774	0.052	109 (47.6)	120 (52.4)	1.167	0.280
21 and above	92 (59)	64 (41)			83 (53.2)	73 (46.8)		

*Gender*								
Male	121 (63.4)	70 (36.6)	16.343	≤0.001^*∗*^	98 (51.3)	93 (48.7)	0.314	0.575
Female	83 (42.8)	111 (57.2)			94 (48.5)	100 (51.5)		

*Religion*								
Hindu	180 (51.3)	171 (48.7)	4.638	0.031^*∗*^	175 (49.9)	176 (50.1)	0.000	0.987
Non-Hindu	24 (70.6)	10 (29.4)			17 (50)	17 (50)		

*Ethnicity*								
Brahmin/Chhetri	137 (52.9)	122 (47.1)	0.003	0.959	129 (49.8)	130 (50.2)	0.001	0.972
Others	67 (53.2)	59 (46.8)			63 (50)	63 (50)		

*Current living status*								
Not living with family	71 (62.3)	43 (37.7)	5.615	0.018^*∗*^	71 (62.3)	43 (37.7)	9.978	≤0.001^*∗*^
Living with family	133 (49.1)	138 (50.9)			121 (44.6)	150 (55.4)		

*Family income*								
Below 50,000	98 (57.3)	73 (42.7)	2.308	0.129	89 (52)	82 (48)	0.583	0.445
50,000 and above	106 (49.5)	108 (50.5)			103 (48.1)	111 (51.9)		

*Body Mass Index*								
Underweight	25 (49)	26 (51)	1.813	0.404	26 (51)	25 (49)	0.749	0.688
Normal	168 (54.5)	140 (45.5)			151 (49)	157 (51)		
Overweight	11 (42.3)	15 (57.7)			15 (57.7)	11 (42.3)		

*Sickness frequency*								
Often	106 (50.2)	105 (49.8)	1.417	0.234	106 (50.2)	105 (49.8)	0.025	0.874
Less often	98 (56.3)	76 (43.7)			86 (49.4)	88 (50.6)		

*Food related health problem within 3 months*								
No	100 (51.5)	94 (48.5)	0.326	0.568	101 (52.1)	93 (47.9)	0.751	0.386
Yes	104 (54.5)	87 (45.5)			91 (47.6)	100 (52.4)		

*Other health problems*								
No	76 (55.5)	61 (44.5)	0.528	0.467	66 (48.2)	71 (51.8)	0.244	0.621
Yes	128 (51.6)	120 (48.4)			126 (50.8)	122 (49.2)		

*Smoke*								
No	159 (49.4)	163 (50.6)	10.284	≤0.001^*∗*^	152 (47.2)	170 (52.8)	5.591	0.018^*∗*^
Yes	45 (71.4)	18 (28.6)			40 (63.5)	23 (36.5)		

*Alcohol*								
No	110 (47.8)	120 (52.2)	6.108	0.013^*∗*^	109 (47.4)	121 (52.6)	1.404	0.236
Yes	94 (60.6)	61 (39.4)			83 (53.5)	72 (46.5)		

*Food items avoided*								
No	117 (53.2)	103 (46.8)	0.008	0.930	120 (54.5)	100 (45.5)	4.488	0.034^*∗*^
Yes	87 (52.7)	78 (47.3)			72 (43.6)	93 (56.4)		

*Additional food intake*								
No	101 (56.1)	79 (43.9)	1.324	0.250	87 (48.3)	93 (51.7)	0.319	0.572
Yes	103 (50.2)	102 (49.8)			105 (51.2)	100 (48.8)		

*Fasting*								
No	131 (54.4)	110 (45.6)	0.485	0.486	111 (46.1)	130 (53.9)	3.745	0.053
Yes	73 (50.7)	71 (49.3)			81 (56.3)	63 (43.8)		

^*∗*^Statistically significant association.

**Table 5:** Logistic regression for food choice and eating practice.

Variables	Good food choice and good eating practice			
	COR (95% CI)^#^	*p* value	AOR (95% CI)^##^	*p* value
*Good food choice*				
Gender				
Female (male)	2.312 (1.535–3.481)	≤0.001^*∗*^	1.738 (1.074–2.812)	0.024^*∗*^

Religion				
Non-Hindu (Hindu)	0.439 (0.204–0.944)	0.035^*∗*^	0.463 (0.211–1.015)	0.054

Current living status				
Living with family (not living with family)	1.713 (1.095–2.680)	0.018^*∗*^	1.387 (0.865–2.224)	0.174

Smoke				
Yes (no)	0.390 (0.217–0.703)	0.002^*∗*^	0.612 (0.308–1.216)	0.161

Alcohol				
Yes (no)	0.595 (0.393–0.899)	0.014^*∗*^	0.888 (0.549–1.436)	0.629

*Good eating practice*				
Current living status				
Living with family (not living with family)	2.047 (1.308–3.204)	≤0.001^*∗*^	2.062 (1.304–3.263)	≤0.001^*∗*^

Smoke				
Yes (no)	0.514 (0.294–0.898)	0.019^*∗*^	0.546 (0.308–0.967)	0.038^*∗*^

Food items avoided				
Yes (no)	1.550 (1.032–2.327)	0.035^*∗*^	1.670 (1.100–2.536)	0.016^*∗*^

( ): reference category; ^*∗*^significant at < 0.05; ^#^COR calculated from the bivariate logistic regression; ^##^AOR calculated from the multivariate logistic regression. *Note.* If in the bivariate analysis the variable was insignificant, it was not included in the multivariate analysis. The multicollinearity was assessed by using VIF (variance infection factor) by using “car” package; all the VIF were less than 5. So, there is no multicollinearity in this model.

## Data Availability

The datasets and materials used and/or analysed during this study can be obtained from the corresponding author upon reasonable request.
